# Dr. C. W. Long, the Discoverer of Anæsthesia

**Published:** 1879-04

**Authors:** 


### DR. C. W. LONG, THE DISCOVERER OF ANÆSTHESIA.

Below we publish the resolutions adopted by the Athens Medical Association, at a meeting held on the 24th ultimo:

That the late Dr. Crawford W. Long, of Athens, Ga., was the true discoverer of anæsthesia there can exist no just or reasonable doubt. Dr. J. Marion Sims, of New York, published in 1877 in the May number of the *Virginia Medical Monthly* a full account of the discovery of anæsthesia, giving all the facts set forth by the different claimants as to the discovery of this great boon to the civilized world. Among these claimants are Dr. C. W. Long, of Athens, Ga.; Dr. Horace Wells, of Hartford, Conn.; Dr. W. T. G. Morton, and Dr. Charles T. Jackson, of Boston, Mass.

Dr. Sims, after giving a plain statement of all the facts bearing upon the question as to who was the true and rightful discoverer of anæsthesia, sums up as follows:

"Long's anæsthesia with sulphuric ether was on the 30th of March, 1842."

"Wells' anæsthesia with nitrous oxide gas was on the 11th of December, 1844."

"Morton's anæsthesia with sulphuric ether was on the 30th of September, 1846."

"Thus we see that Long antedates Wells two years and eight months, and antedates Morton four years and six months.

Morton and Jackson, it seems, were associated together in the medical profession in the city of Boston, and their claims are made conjointly.

It appears from Dr. Sims' publication of facts that Dr. Morton had, upon his claim of being the first to use sulphuric ether for the purpose of producing anæsthesia, applied to Congress for an appropriation which he demanded for his discovery.

Upon this point Dr. Sims says:

"When Long's claim to the honor of discovering anæsthesia was presented to Congress by the Hon. Mr. Dawson, Senator from Georgia, it was formidable enough to block the movements of Morton to get the appropriation he demanded for his discovery. They were so strong that Dr. Charles T. Jackson went to Athens, Georgia, expressly to see Dr. Long on this subject. In a communication to the Boston *Medical and Surgical Jonrnal* April 11th, 1861, Dr. Charles T. Jackson says he visited Dr. Long at Athens, Georgia, on March 8th, 1854, to examine into Dr. Long's claims to being the first to use sulphuric ether as an anæsthetic in surgery, and he further says: "From the documents shown me by Dr. Long it appears that he employed sulphuric ether as an anæsthetic agent—

"*First.* On March 30th, 1842, when he extirpated a small glandular tumor from the neck of James M. Venable, a boy [Mr. Venable was over 21 years old when the operation was performed.—J. M. S.] in Jefferson, Georgia, now dead.

"*Second.* On July 3d, 1842, in the amputation of the toe of a negro boy belonging to Mrs. Hemphill, of Jackson, Georgia.

"*Third.* On September 9th, 1843, in the extirpation of a tumor from the head of Mary Vincent, of Jackson, Georgia.

"*Fourth.* On January 8th, 1845, in the amputation of a finger of a negro boy belonging to Ralph Baily, of Jackson, Georgia.

"Copies of the letters and depositions proving these operations with ether were all shown me by Dr. Long.

He also referred me to physicians in Jefferson who knew of the operations at the time."

The above extract from Dr. Jackson's paper to the Boston *Medical Journal* recognizes Long's claim to being the first to produce anæsthesia for surgical operations, but it does not tell the whole story of Dr. Jackson's visit to Dr. Long.

Dr. Long has furnished me with all the evidence, consisting of affidavits, certificates, book entries, etc., that Dr. Jackson examined. He had also written me fully on the subject, and every fact that I have stated can be sustained by documentary evidence.

In one of Dr. Long's letters to me (Nov. 5, 1876), he says:

"In 1854 Dr. Charles T. Jackson came to Georgia and spent two days with me in Athens, most of the time in my office, examining books, accounts, dates and certificates establishing the time, etc., of my operations. He expressed himself satisfied with the correctness of my claim to the first use of ether as an anæsthetic in surgical operations. Dr. Jackson informed me that he would go from Athens to Dahlonega, Ga., and as I knew he must pass through Jefferson, where I resided up to 1850, and where my first operations under ether were performed, I requested him to stop in Jefferson and see some of the physician there who witnessed or knew of the operations, and also a number of the citizens of the village who either witnessed the operations or were familiar with them from common report. Dr. Jackson spent one or more days in Jefferson, and on his return, expressed himself satisfied with the testimony.

"In Dr. Jackson's communication to the Boston *Medical and Surgical Journal* (April 11th, 1861), he neglected to say anything of the information he obtained while in Jefferson, although he admitted to me on his return that the evidence was perfectly satisfactory."

The Hon. C. W. Andrews, of Madison, Ga., informs me that he was in Dr. Long's employ and in his office when Dr. Jackson spent a whole day with Long in comparing notes and talking over the subject of etherization, and it seems that the real object of Dr. Jackson's visit to Dr. Long was to induce Long to unite with him in laying their conjoint claims before Congress as the real discoverers of anæsthesia as opposed to those of Morton. Jackson was willing to concede to Long the honor of being the first to use ether in surgical operations, but wished Long to concede to him the honor of priority in making the discovery of the principle of anæsthesia when he inhaled ether to relieve the pain and difficulty of breathing after inhaling chlorine gas (as Sir Humphrey Davy had done before.)

Dr. Long says (February 8th, 1877): "In our conversation I understood Dr. Jackson to yield the point of priority to me—and so did the Hon. C. W. Andrews. I did not admit to him that he was the first to make the discovery

—leaving to me its practical application; and when he proposed to me to unite our claims—he to claim the discovery and I its first practical use in surgical operations—I positively refused. I was satisfied that I was entitled to the credit of the discovery, as well as of the first practical use of ether in surgical operations.

"Instead of writing to Senator Dawson to unite our claims as Dr. Jackson requested, I wrote to Mr. Dawson to make no such compromise, but to place my claims solely on their merits; and if you will consult the Congressional proceedings of that time you will see that Mr. Dawson presented my claims separate and independent.'"

Dr. Jackson is further quoted by Dr. Sims as having said to Dr. Long during his visit to Athens in March, 1854, "You have the advantage of priority in date and in the first use of ether as an anæsthetic; but we have the advantage of priority of publication."

Now, upon this point Dr. Jackson is evidently mistaken as to his "advantage of priority of publication." For abundant and indubitable evidence is given that Dr. Long did exhibit to medical men and to the community at large his operations under the influence of ether in 1842, while Wells, Jackson, and Morton, made no exhibit until as late as 1844 and 1846. It is true Dr. Long may not have published his discovery in the medical journals of the country, nor does it appear that the other claimants did, but exhibiting their experiments in the large cities of New York and Boston, of course better opportunities were afforded for disseminating the facts throughout the medical world. Abundant evidence has been produced by Dr. Long to prove that he made no secret of his discovery, but on the contrary, communicated it as rapidly to the medical fraternity as his restricted and limited facilities would permit, and the fact that he perhaps did not publish it through the medical journals of the country, makes him none the less the true discoverer.

Having submitted sufficient proof from the records to establish beyond controversy the fact that Dr. Long was the first ever to use sulphuric ether as an anæsthetic agent in surgical operations, and which fact has been fully admitted and substantiated by the very testimony of the other claimants themselves, and especially by the evidence laid before Congress by Hon. W. C. Dawson, as to Dr. Long being the true discoverer, we now come to the real purpose of this notice.

What, let us ask ourselves, has been the great blessing bestowed upon the civilized world by this greatest of all human discoveries? While the appreciative spirit of the world is now being so extensively exercised to properly honor and immortalize the names and fame of those who have so adorned the nineteenth century with inventions and discoveries of such inestimable value to science as applied to the more rapid growth and development of our material interests, yet let it be remembered that to Dr. Crawford W. Long, belongs the honor of having discovered a blessing to mankind incomparably greater than all the combined discoveries of this or any other age.

A Newton, a Gallileo, a Fulton, a Morse and various other devotees of science have, through their invaluable contributions to the scientific, commercial and industrial world rendered their names as lastingly honored as time itself. A Harvey, a Jenner, a Cooper, a Daennec, and indeed many earnest and devoted followers of the science of medicine have most enduringly inscribed their names upon the roll of the world's benefactors, but to Dr. Long was reserved the honor of discovering the greatest of all boons to suffering man; that which so quiets to rest the nervous sensibilities of our natures as to render us insensible to the many ills and sufferings of life, and to make safe and endurable the frightful operations of the surgeon's hand, so often demanded through accident and disease.

Wheresoever science and civilization have marked their impress upon this habitable globe, bestowing in their rapid and onward march their benefits and blessings, there will we find the immeasurable benefactions which have been vouchsafed to suffering mortals through the discovery of anæsthesia. Dr. Long, not by accident, but in a most philosophical and logical manner, and with that modesty and skillful care which come of true science and learning, did, through the earnest and scientific application of that higher order of mind with which he was endowed, nobly and successfully fill his sphere in life, and, in so doing, has justly and truly won for himself the proud honor of being one of the world's benefactors, and which honor will be as imperishable as the blessings which have and which will ever continue to come of his invaluable discovery. Appreciating, as a true Christian gentleman and philanthropist, and as a true man of science, the sphere of usefulness which he was called upon to fill through the kind and most benevolent endowments of nature, he was ever earnest and assiduous in the application of his skill and his talents to the amelioration of the sufferings of his fellow-man. How pleasing is the reflection that, with this true man of science, this Christian and philanthropist, a life of such usefulness should have been so beautifully and so honorably ended. He died at his post, and almost the last request of his life was that he might be permitted to relieve the suffering of his patient through the use of that agent, the discovery of which made him one of the greatest among the living, and now one of the most honored and renowned among the dead, men of medicine. It is a fact which should fill alike with pride the heart of every medical man in Georgia and in America, that this great discover was a native of our State and our country.

Congress haying as yet failed to make that appropriation in behalf of this discovery. and which it so richly and so justly demands, then it should be the proud honor, and the most pleasing duty, of the medical profession of Georgia, of America, and indeed of the whole world, to make a just and merited recognition and tribute of honor to the memory of the great benefactor. We would suggest, in this connection, that, through the medical profession, a monument as enduring as brass should be erected upon American soil in honor of the discoverer of anæsthesia. It is eminently appropriate that the Medical Association of Athens, the home of Dr. Long's adoption, should have taken the initiatory in this work of justice, this labor of love. And we trust the State Medical Association will most unanimously and enthusiastically second the movement, and that, through this Association the American Medical Association, which is to meet in the city of Atlanta this summer, may be induced to inaugurate such steps as will speedily result in honor and justice to the name and memory of Dr. Crawford W. Long, of Athens, Ga., the true and recognized discoverer of anæsthesia.

*Rssolutions.*—Whereas, the recent compilation of facts by Dr. J. Marion Sims, of New York, in regard to the discovery of anæsthesia, clearly proves Dr. Crawford W. Long to be the first to ever use sulphuric ether as an anæsthetic agent in surgical operations; and,

*Whereas,* Dr. Long was a native Georgian, and especially a native of our own section, we, the Medical Association of Athens, and of which he was the honored and esteemed President, must necessarily feel much pride in claiming so valuable, so useful and so scientific a man as our companion in medicine, and likewise feeling that not only a most pleasing sense of duty, but a most justifiable sense of pride and honor, make it eminently proper and commendable that this Association should take the initiatory steps in bringing about, on the part of the medical profession, a full and just recognition of the claims of Dr. Long to being the first and true discoverer of anæsthesia; therefore, be it

*Resolved,* That from the accumulation of facts and evidence deduced, this Society is fully satisfied that Dr. Crawford W. Long, of Athens, Ga., was the first person who ever used sulphuric ether as an anæsthetic agent, in surgical operations; and as an act of justice to the distinguished discoverer, now deceased, and to the honor of the profession of our own State, we most respectfully recommend and request that the Georgia Medical Association, soon to hold its annual session in the city of Rome, take such steps as shall definitely determine Dr. Long's claim to priority in the use and discovery of anæsthesia; and that the State Medical Association be further requested to bring this matter before the American Medical Association, which is to hold its next annual session in the city of Atlanta during the approaching summer, that through this national convention of medical men Dr.

Long's claim may be established throughout the civilized world.

*Resolved*, That a copy of these resolutions be forwarded to the President and Secretary of the State Medical Association, and also to the different medical journals of our State, with the request that they publish the same.

| | |
|---|---|
| H. Hull, M.D. | J. B. Carlton, M.D. |
| W. A. Carlton, M. D. | John Gerdine, M.D. |
| G. L. McCleskey, M.D. | J. E. Pope, M.D. |
| Wm. King, M.D. | H. H. Carlton, M.D. |

[*Southern Banner*.

